# Lanthionine Ketimine Ethyl Ester Induces Proliferation and Maturation and Regulates Calcium Flux in Primary Mouse Oligodendrocyte Progenitor Cells

**DOI:** 10.1002/jnr.70061

**Published:** 2025-06-27

**Authors:** Veronica T. Cheli, Swathi G. Tumuluri, Zachary McDonald, Travis T. Denton, Jeffrey L. Dupree, Pablo M. Paez, Douglas L. Feinstein

**Affiliations:** ^1^ Institute for Myelin and Glia Exploration, Department of Pharmacology and Toxicology University at Buffalo Buffalo New York USA; ^2^ Department Anesthesiology University of Illinois Chicago Illinois USA; ^3^ Department of Pharmaceutical Sciences, College of Pharmacy & Pharmaceutical Sciences Washington State University Health Sciences Spokane Spokane Washington USA; ^4^ Department of Translational Medicine and Physiology, Elson S. Floyd College of Medicine Washington State University Health Sciences Spokane Spokane Washington USA; ^5^ Steve Gleason Institute for Neuroscience Washington State University Health Sciences Spokane Spokane Washington USA; ^6^ Department of Anatomy and Neurobiology Virginia Commonwealth University Richmond Virginia USA; ^7^ Research Service Richmond VA Medical Center Richmond Virginia USA; ^8^ Jesse Brown VA Medical Center Chicago Illinois USA

**Keywords:** calcium channels, calcium imaging, lanthionine, myelin, NMDA receptors, oligodendrocyte

## Abstract

Previous studies have shown that lanthionine ketimine ethyl ester (LKE), a semi‐synthetic derivative of the endogenous amino acid lanthionine, can induce proliferation and maturation of oligodendrocyte progenitor cells (OPCs) in vivo. In the current study, we examined the effects of LKE on Ca^2+^ influx in primary mouse OPCs, as intracellular Ca^2+^ can regulate those processes. Treatment with LKE stimulated proliferation of OPCs and increased the number of Olig2+, CC1+, and PLP+ cells. LKE also reduced cell death (caspase‐3 expressing cells). Measurements of Ca^2+^ flux showed that LKE increased basal Ca^2+^ levels, reduced Ca^2+^ influx following stimulation with glutamate or ATP, and increased Ca^2+^ flux because of depolarization with KCl. Reduced Ca^2+^ responses were also observed following treatment with a peptide that disrupts interactions of collapsin response mediated protein 2 (CRMP2), a primary target of LKE. These findings demonstrate regulation of Ca^2+^ levels in OPCs by LKE and suggest that these actions may be mediated, in part, by CRMP2. LKE or related analogs could therefore be of benefit for the treatment of multiple sclerosis as well as other demyelinating conditions.

## Graphical abstract

LKE increased OPC proliferation and maturation. LKE reduced Ca responses to ATP and glutamate, and increased responses to KCl. Similar effects occurred with a peptide that disrupts Collapsin Response Mediator Protein 2 interactions with NMDARs and VGCCs.

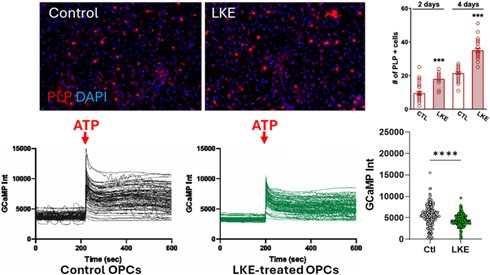

**Summary**

- The amino acid derivative lanthionine ketimine ethyl ester (LKE) increases oligodendrocyte (OLG) numbers and maturation in animal models of multiple sclerosis; however, the mechanisms involved are not known.
- Here we confirm that LKE increases OLG proliferation and differentiation using cell cultures and show that LKE regulates intracellular calcium levels in these cells.
- Similar results were obtained when interactions of CRMP2, a protein target of LKE, with NMDARs and Voltage Gated Calcium Channels were inhibited.
- These findings suggest LKE could accelerate remyelination by increasing OLG numbers and function via suppression of CRMP2 function and modulation of calcium responses.

## 1 Introduction

Lanthionine ketenamine ethyl ester, often incorrectly referred to as lanthionine ketimine ethyl ester (LKE) is a semi‐synthetic derivative of the naturally occurring amino acid lanthionine (Hensley et al. 2010). LKE was shown to have neuroprotective actions in a variety of models of neurodegenerative diseases and conditions including Parkinson's disease (PD) (Togashi et al. 2020; Yazawa et al. 2022), Alzheimer's disease (AD) (Hensley et al. 2013; Koehler et al. 2018), spinal cord injury (Kotaka et al. 2017), and MS (Dupree et al. 2015). LKE also shows protective actions in primary cultures of neurons (Gonzalez Porras et al. 2023; Marangoni et al. 2018) and oligodendrocytes (Savchenko et al. 2019). In previous studies, we demonstrated that LKE shows beneficial actions in mouse models of multiple sclerosis (MS). In experimental autoimmune encephalomyelitis (EAE, a commonly used model for MS), daily administration of LKE reduced clinical scores and neuroinflammatory activation in the cerebellum (Dupree et al. 2015). We subsequently showed that LKE accelerated remyelination following chemical demyelination with the copper chelator cuprizone (Dupree et al. 2022). In that study, after 2 weeks of remyelination, LKE increased the percentage of remyelinated axons from 50% to 67%, and the ratio of axon diameter to fiber diameter (the g‐ratio, an index of axonal capacity to transmit signals) was restored to near control values. These changes were associated with an increase in the number of oligodendrocyte (OLG) progenitor cells (OPCs) and mature OLGs, suggesting effects of LKE on OPC proliferation, survival, and maturation. Similar effects on OPCs occurred using primary OPC cultures, suggesting direct actions of LKE on these cells (Savchenko et al. 2019).

The mechanisms of action of LKE are not fully defined; however, proteomics analysis showed that LKE binds to collapsin response mediator protein 2 (CRMP2), a structural protein that interacts with protein targets including tubulin (Hensley et al. 2010) and various synaptic proteins (Stratton et al. 2020). Subsequent studies showed that LKE disrupts interactions between CRMP2 and several of its binding partners including the N‐methyl‐D‐aspartate receptor (NMDAR) NR2B (Brittain, Chen, et al. 2011) and NR2A subunits, and the N‐type voltage‐gated calcium channel (VGCC) CaV2.2 (Chi et al. 2009). Disruption of CRMP2:NMDAR interactions using small peptides reduced excitotoxic damage in focal cerebral ischemia and traumatic brain injury (Brittain, Chen, et al. 2011; Brittain et al. 2012). Similarly, disruption of CRMP2:CaV2.2 interactions using small CRMP2‐derived peptides reduced nociceptive responses (Brittain, Duarte, et al. 2011; Gomez et al. 2023; Perez‐Miller et al. 2024). Although LKE has not yet been demonstrated to modulate CaV2.2 activity, the addition of LKE to primary neurons reduced glutamate‐induced toxicity (Marangoni et al. 2018).

In view of findings that LKE can reduce excitotoxicity, and modulate Ca^2+^ flux; and since these events are critical to OPC survival (Matute 2011; Micu et al. 2006) and maturation (Cheli et al. 2016, 2015; Santiago Gonzalez et al. 2017), we hypothesized that LKE directly alters these responses in OPCs. In the current study we show that Ca^2+^ influx due to glutamate or ATP is reduced by LKE, whereas influx due to KCl‐induced depolarization is increased. Quantitation of mRNAs suggests that some of these effects on Ca^2+^ flux are associated with changes in mRNAs encoding relevant channels and receptors. Similar findings were observed following incubation of OPCs with a synthetic peptide which disrupts CRMP2 interactions with target proteins, suggesting that some effects of LKE on Ca^2+^ influx are mediated through CRMP2.

## 2 Methods and Materials

### 2.1 Animals

C57Bl6 mice were obtained from Charles River Laboratories. All animal studies were approved by the Institution Animal Care and Use Committees at the University of Illinois, University of Buffalo, and Jesse Brown and Richmond VA Medical Centers.

### 2.2 Synthesis of LKE

LKE was synthesized as described (Dupree et al. 2022). A solution of ethyl cysteine hydrochloride (1.48 g, 8.01 mmol) in water (8 mL) was added to a stirring solution of bromopyruvate (1.56 g, 8.41 mmol) in water (10 mL) over 30s. After formation of a solid, the flask was purged with argon, stirred overnight, then the white solid was collected by vacuum filtration (Buchner funnel, #4 filter paper) and residual volatiles removed under high vacuum. LKE (1.36 g, 78.24% yield) was recovered as a white solid and determined to be > 99% pure by 1H NMR analysis.

### 2.3 Primary Cultures of OPCs

Primary cultures of cortical OPCs were prepared as described (Cheli et al. 2015, 2023; Wan et al. 2020), which results in > 98% OPCs and < 1% GFAP stained astrocytes or Iba1 stained microglia. Cerebral hemispheres from 1‐day old mice were mechanically dissociated and then plated in poly‐D‐lysine‐coated flasks in DMEM/F12 (1:1 v/v) (Invitrogen), supplemented with 10% fetal bovine serum (FBS) (Life Technologies). After 4 h, the medium was changed and cells grown in DMEM/F12 supplemented with insulin (5 μg/mL), apotransferrin (50 μg/mL), sodium selenite (30 nM), d‐Biotin (10 mM), and 10% FBS (Life Technologies). Every 3 days 2/3 of the media was changed. OPCs were purified from mixed glia after 14 days by a differential shaking and adhesion procedure. The detached cells were plated into Petri dishes for 30 min at 37°C to allow microglia and astrocytes to adhere, and then non‐attached cells were collected and plated on poly‐D‐lysine‐coated coverslips in DMEM/F12 supplemented with insulin (5 μg/mL), apotransferrin (50 μg/mL), sodium selenite (30 nM), 0.1% BSA, progesterone (0.06 ng/mL), putrescine (16 μg/mL) (Sigma), and 2% FBS. OPCs were kept in mitogens platelet derived growth factor (PDGF), and basic fibroblast growth factor (bFGF, 20 ng/mL) (Peprotech) for 2 days and then induced to differentiate by mitogen withdrawal and addition of tri‐iodothyronine (T3, 50 nM). Since litter size can affect pup development, litters with less than 5 or more than 10 pups were not used.

### 2.4 Immunocytochemical Staining

After 2 days in proliferation media, the cells were washed, then fresh proliferation media lacking mitogens containing 0 or 50 μM LKE added. After 2 or 4 days, the OPCs were stained with antibodies against several OLG markers and examined by confocal microscopy. In brief, the cells were rinsed in PBS and fixed in 4% buffered paraformaldehyde for 20 min at room temperature. After rinsing in PBS, the cells were permeabilized with 0.1% Triton X‐100 in PBS for 2 min at room temperature and then processed for immunocytochemistry following the protocol outlined previously (Cheli et al. 2016). The fixed cells were incubated in blocking solution (5% goat serum in PBS), followed by overnight incubation at 4°C with primary antibodies. The cells were then incubated with appropriate secondary antibodies (1:400; Jackson ImmunoResearch), nuclei were stained with the fluorescent dye DAPI (Life Technologies), mounted onto slides with Aquamount (Thermo Scientific), and fluorescent images were obtained using a spinning disc confocal microscope (Olympus, IX83‐DSU). Cells were counted semi‐automatically using MetaMorph software (Molecular Devices) in 10 randomly selected fields per well, using a 10× objective per 0.3 mm^2^ field, resulting in counts of > 4000 cells per well. Quantitative analysis was done in two independent wells per experimental condition by counting the antigen‐positive and DAPI‐positive cells (e.g., the total number of cells). The numbers of DAPI‐stained nuclei were homogeneous across experimental conditions ranging from 350–400 cells per field after 2 days and from 400–450 cells per field for 4 days. Data represent pooled results from at least two independent cultures. Quantitation was performed by an investigator blinded to the experimental conditions. The primary antibodies used for immunocytochemistry were against caspase‐3 (mouse; 1:400; Cell Signaling, 96,615), the anti‐adenomatous polyposis coli (APC) clone CC1 (mouse; 1:300; EMD Millipore, OP80), the marker of proliferation Ki67 (mouse; 1:200; BD Bio‐sciences, AB_393778), the proteoglycan neuron‐glial antigen 2 (NG2, rabbit; 1:400; END Millipore, AB_5320), the OLG transcription factor Olig2 (rabbit; 1:500; EMD Millipore, AB_9610), and proteolipid protein (PLP, rat; 1:250, AA3‐PLP/DM20).

### 2.5 RNA Quantitation

For RNA analysis, OPCs were induced to differentiate by mitogen withdrawal and addition of T3 for 24 h, then 10 μM LKE was added, cells were incubated for 2 days, and then RNA was isolated using Trizol (Zymo Research) according to instructions. Total RNA (1 μg) was converted to cDNA using High‐Capacity cDNA Reverse Transcription Kit (ThermoFisher #4368814). The cDNA was amplified with specific primers (Table 1) using FastStart Universal SYBR Green Master mix (Applied Biosystems, #04913914001) in a BioRad CFX96 PCR machine (BioRad, Hercules, CA). Relative mRNA levels were calculated from cycle take‐off and normalized to values measured for β‐actin in the same samples.

**TABLE 1:** PCR primers.

Target	Forward	Reverse
Kir3.3	GAGGAACTGGAGATTGTGGTC	AGCACGTTTAAGGTCGGAAG
Kv1.1	ATCAGAACTGGTAACTGCACC	AGTATCTACAGAGCGGGACAG
CaV2.1	AGGCTGGAATTAAGATCGTGG	CCTCAGTGTCCGTAGATCAAAC
CaV2.2	CCACAAACCTGACGAGATGAC	GAGGATGGAACAGGGAAACAG
Cav2.3	AAAGCTCGTGTCAGGAATACC	AACTTGCCACTGTAGAACTCC
GluN2b	AAGGAGAGGAAGTGGGAGAG	AAGGTAACGATGCTCAGATGG
GlunN2a	TGGTGATCGTGCTGAATAAGG	AGGTGACAATGCTGAGGTG
GABARa5	GGGAATGGACAATGGAATGC	TGTCATTGGTCTCGTCTTGTAC
P2Y6	TGTGTCAGAGGGAGTTTTCAG	TGGCTTTTAGTTTACCCCTGTG
mAChR3	TGGACTGTGGATTGAGTGAAC	GCCCTTATTGTACCTTTGCTG

### 2.6 Calcium Imaging

Primary cultures of cortical OPCs were prepared as described above. One day after plating, OPCs were infected with Ad‐GCaMP6 (Vector labs #1910) at a multiplicity of infection (MOI) of 100 viruses per cell and incubated for 24 h. The following day, OPCs were treated with LKE (50 μM) for 24 h or 48 h. Before imaging, OPCs were washed in serum‐ and phenol red‐free DMEM, incubated for 25 min at 37°C and 5% CO_2_, washed 4 times in DMEM, and stored in DMEM for 10 min before being imaged. Ca^2+^ influx and resting Ca^2+^ levels were measured in serum‐ and phenol red‐free HBSS containing 1.3 mM Ca^2+^ and 1 mM Mg^2+^. In some experiments, Ca^2+^ influx was measured by incubating OPCs for 25 min in serum‐ and phenol red‐free DMEM containing 4 μM fura‐2 (AM) (Life Technologies) plus 0.08% Pluronic F127 (Life Technologies). Cells were then washed four times in DMEM and stored in DMEM for 10 min before being imaged. GCaMP was excited at 920 nm and fura‐2 by alternating 340 and 380 nm. Fluorescence signals were acquired every 2 s by means of a high‐speed wavelength‐switching device (Sutter Instruments, Lambda DG4). A spinning disc confocal inverted microscope (Olympus, IX83‐DSU) equipped with a CCD camera (Hamamatsu, ORCA‐R2) measured fluorescence. Ca^2+^ influx and resting Ca^2+^ levels were measured on individual cell bodies using the image analysis software Meta Fluor (Molecular Devices). At least 200 cells for each experimental condition were analyzed. To minimize bleaching, excitation light intensity and sampling frequency were kept as low as possible.

### 2.7 Data Analysis

Group averages were compared by ordinary 1‐way ANOVA, and Tukey's post hoc comparison. Two samples were compared by parametric *t*‐tests. Outliers were identified using Rout and Grubb's methods. Values shown for peak Ca^2+^ levels in Figures 3, 4, 5 have basal Ca^2+^ levels subtracted. Comparisons were made using GraphPad Prism 10.5 (GraphPad) and significance was taken at *p* < 0.05.

## 3 Results

**FIGURE 1:**
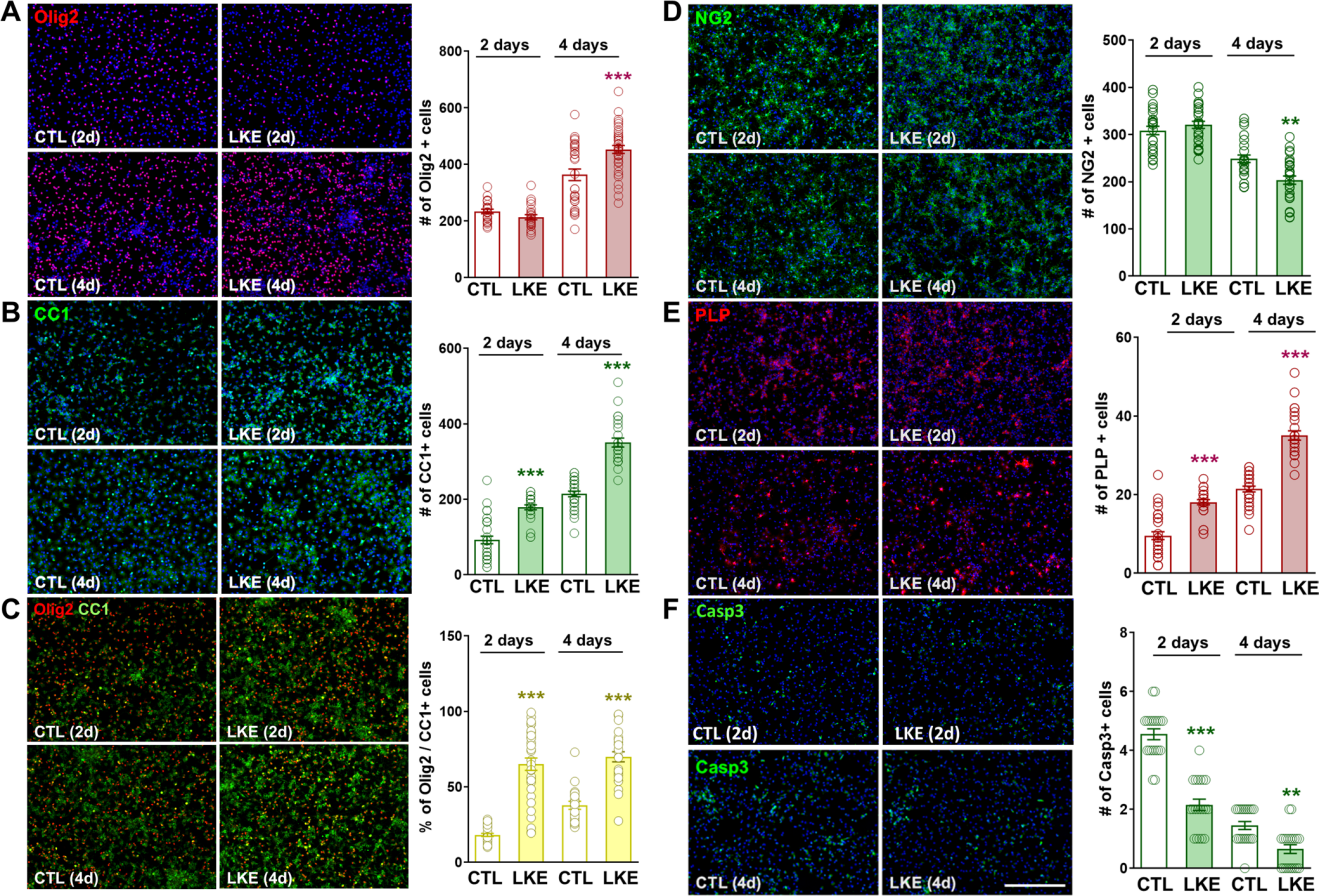
LKE increases OPC maturation. Primary mouse OPCs were incubated with 50 μM LKE for 2 or 4 days, after which the cells were fixed and processed for immunocytochemical staining with the indicated antibodies. Representative images are shown for (A) Olig2; (B) CC1; (C) Olig2 plus CC1; (D) NG2; (E) PLP; and (F) Caspase‐3. The average number of cells per field of view is shown. The % Olig2+/Ki67+ cells is the % of the Olig2+ cells that co‐express CC1. **p < 0.05; ***p < 0.005 versus same day control cells, 1‐way ANOVA, Tukey's post hoc comparisons. The scale bar shown in panel F is 90 μm and is the same for all panels.

We first examined the effects of LKE on OPC proliferation and maturation. After 2 days incubation with LKE, immunostaining revealed no change in the number of Olig2+ cells (Figure 1A) and a significant increase in the number of CC1+ cells (Figure 1B). Double‐immunostaining shows that LKE increased the % of Olig2+ cells that co‐expressed CC1 from 18% to about 65% (Figure 1C). LKE did not alter the number of NG2+ cells (Figure 1D) but increased the number of PLP+ cells (Figure 1E). We also observed a significant decrease in the number of Caspase‐3+‐stained cells (Figure 1F). After 4 days incubation, the number of Olig2+, CC1+, and PLP+ cells further increased because of the presence of LKE; and the % of double‐labeled Olig2+/ CC1+ cells was increased compared to control cells but remained similar to the increase observed after 2 days. Consistent with increased OPC maturation, immunostaining for NG2 was reduced by LKE, and there was a further decrease in caspase‐3 staining pointing to a cyto‐protective effect of LKE.

**FIGURE 2:**
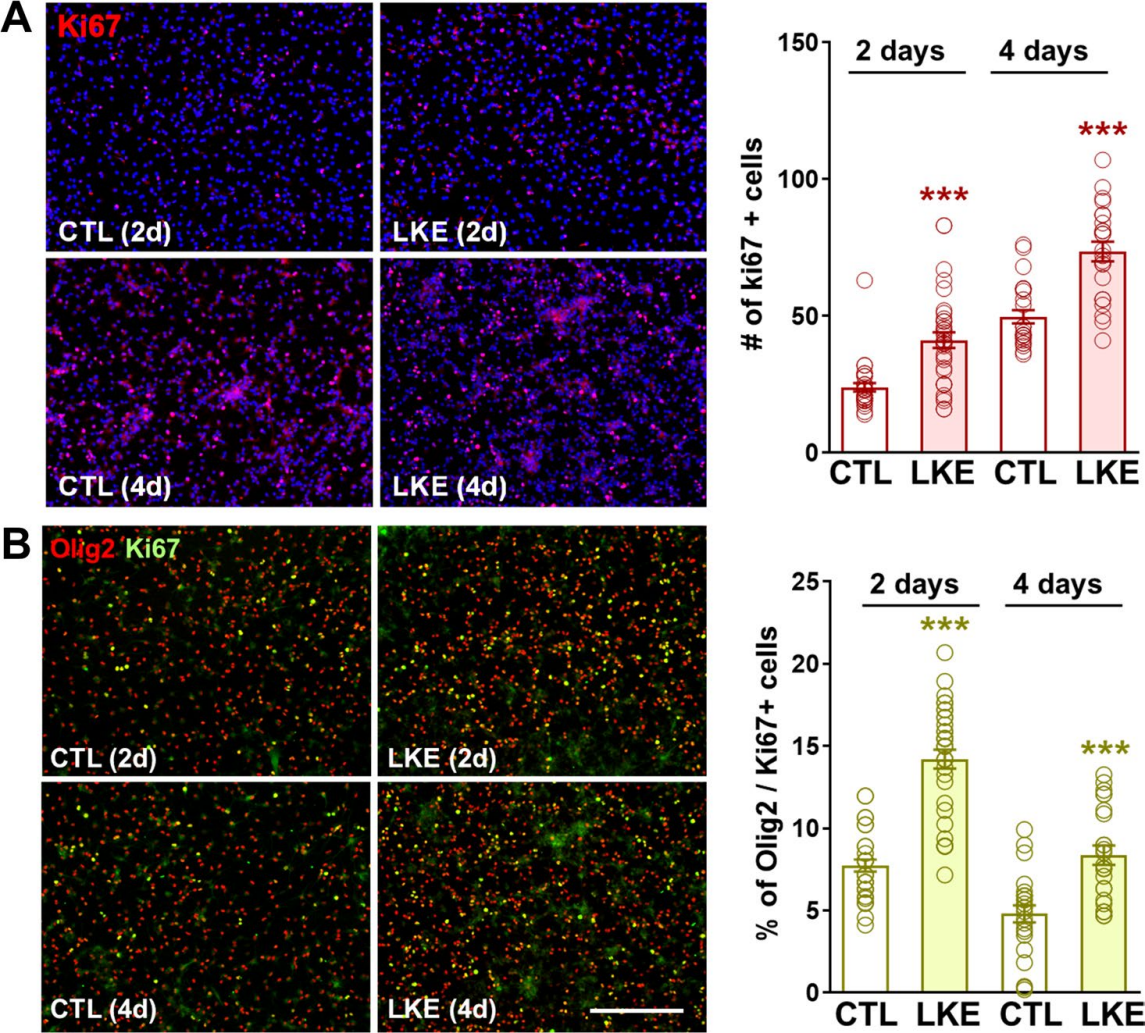
LKE increases OPC proliferation. Primary mouse OPCs were incubated with 50 μM LKE for 2 or 4 days, after which the cells were fixed and processed for immunocytochemical staining. (A) Representative images for Ki67 and the number of stained cells per field of view. (B) Representative images showing co‐labeling of Ki67 with Olig2. The % Olig2+/Ki67+ cells is the % of the Olig2+ cells that co‐express Ki67. ***p < 0.005 versus same day control cells, 1‐way ANOVA, Tukey's post hoc comparisons. The scale bar shown in panel B is 90 μm and is same for all panels.

To determine if LKE increased OPC maturation, we stained cells for the proliferation marker Ki67. Incubation with LKE significantly increased the number of Ki67+ stained cells after both 2 and 4 days incubation (Figure 2A). Double‐immunostaining (Figure 2B) shows that LKE increased the % of Olig2+ cells that co‐express Ki67 from 7% to 14% after 2 days; and from 4% to 8% after 4 days, consistent with a proliferative effect of LKE on a sub‐population of OPCs.

**FIGURE 3:**
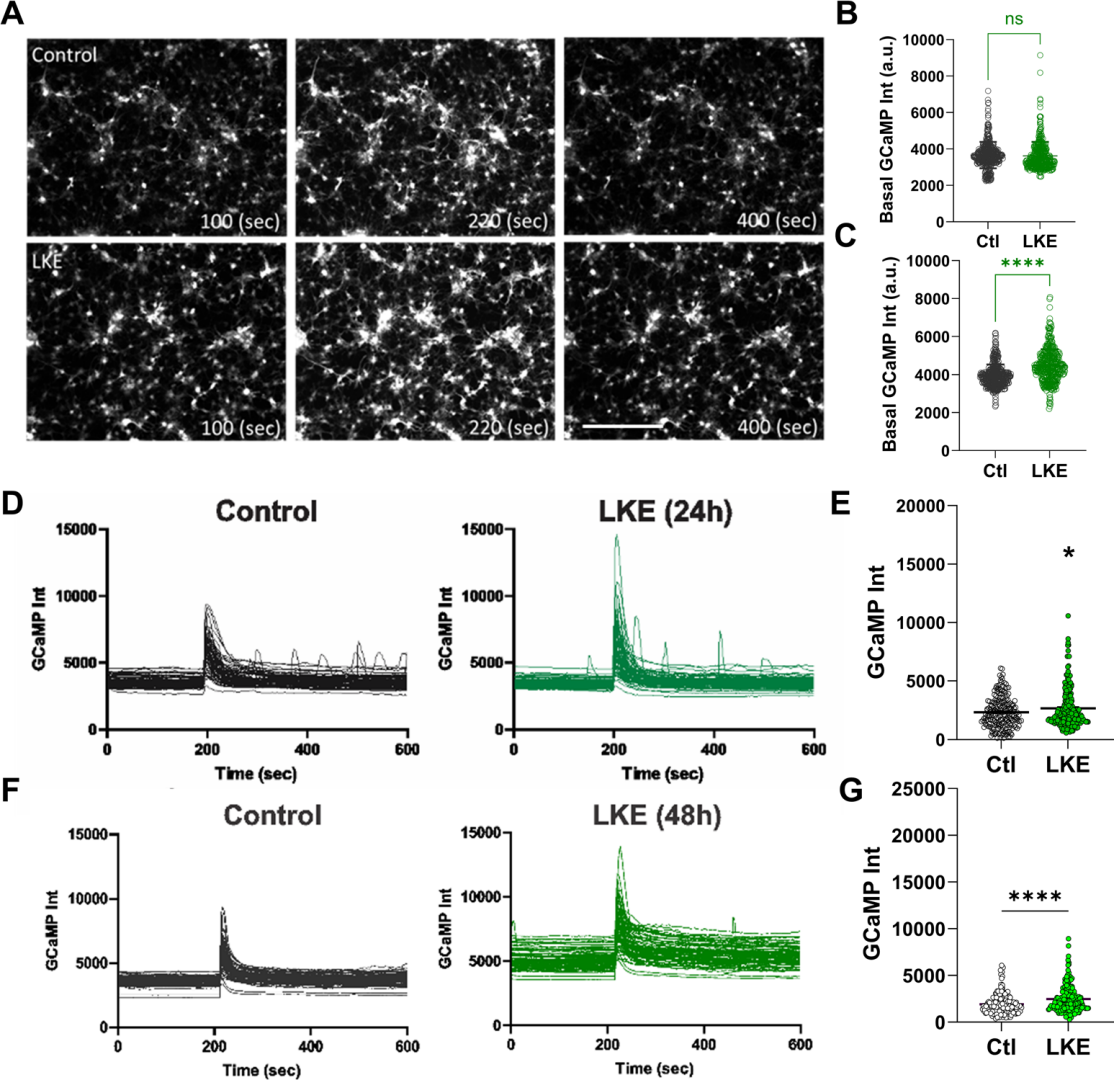
OPC Ca^2+^ responses to KCl are reduced by LKE. OPCs were infected with Ad‐GCaMP6 for 1 day, then incubated with 50 μM LKE for 24 or 48 h, and imaged before and after stimulation with 50 mM KCl. GCaMP images were obtained at 2 s intervals for a total of 10 min. (A) Representative images show basal, peak, and post peak fluorescence. Average basal Ca^2+^ levels after (B) 24 and (C) 48 h with LKE. Individual cell traces measured after (D) 24 and (F) 48 h. Average peak net Ca^2+^ levels (after subtraction of basal levels) measured (E) 24 and (G) 48 h after LKE. Data are mean ± SE of 200 individual cells. *p < 0.05; ****p < 0.0001 versus same day control cells, unpaired *t*‐test. Scale bar is 80 μm.

Since intracellular Ca^2+^ can influence OPC proliferation, survival, and maturation, we examined the effects of LKE on Ca^2+^ flux in OPCs by monitoring the change in fluorescence of GCamp6. OPCs were incubated with LKE for 24 or 48 h, transfected with Ad‐GCamp6, then Ca^2+^ levels monitored over the next 10 min following stimulation (Figure 3). Measurements made prior to stimulation show that LKE did not affect basal Ca^2+^ levels after 24 h (Figure 3B), while causing a modest increase (approximately 13%) after 48 h (Figure 3C). Depolarization with 50 mM KCl significantly increased the Ca^2+^ amplitude by 1.6‐fold after 24 h (Figure 3D) and 1.5‐fold after 48 h (Figure 3F), and those changes were further increased in the LKE‐treated cells by 14% after 24 h (Figure 3E) and 29% after 48 h (Figure 3G).

**FIGURE 4:**
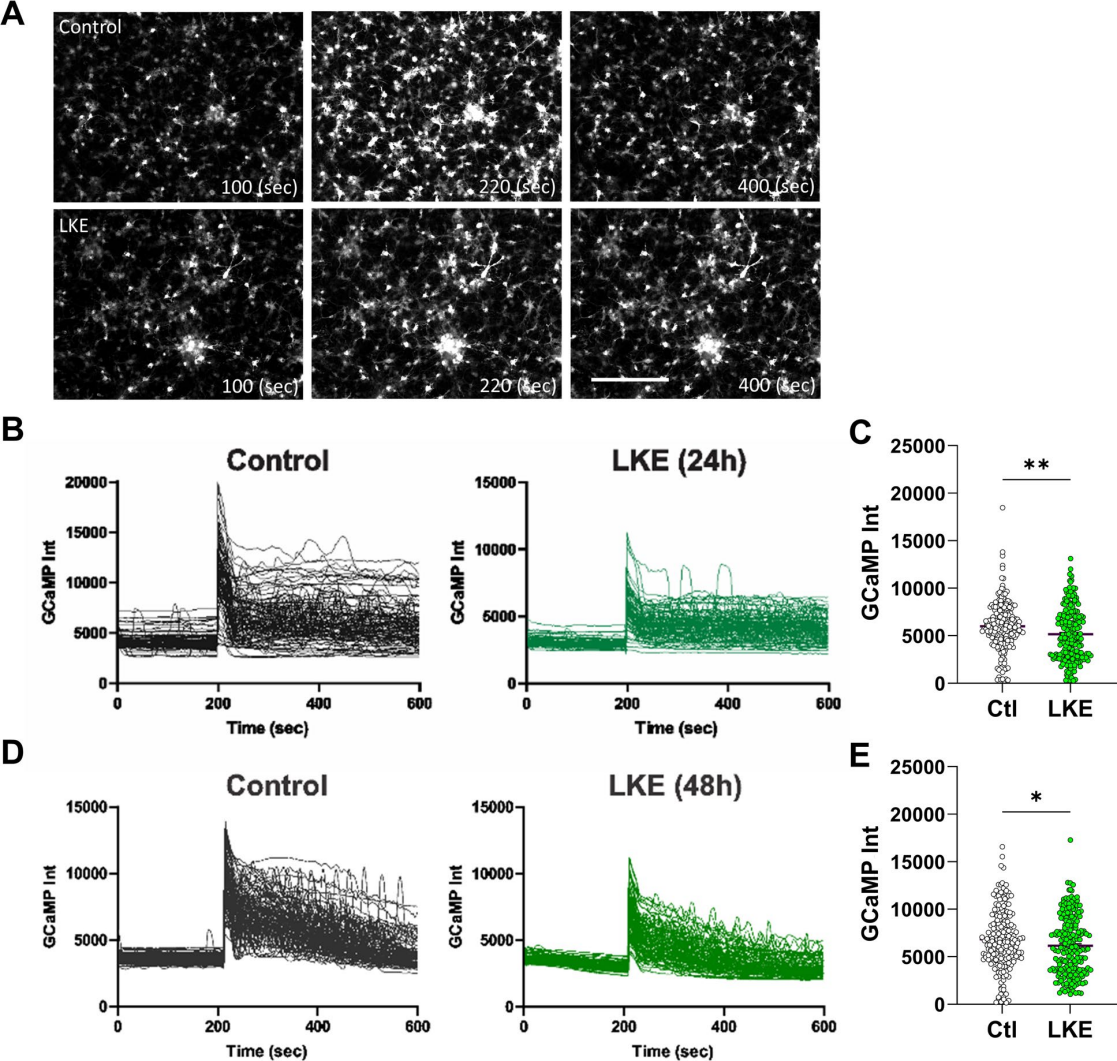
OPC Ca^2+^ responses to glutamate are reduced by LKE. OPCs were infected with Ad‐GCaMP6 for 1 day, then incubated with 50 μM LKE for 24 or 48 h, and imaged before and after stimulation with 100 μM glutamate. GCaMP images were obtained at 2 s intervals for a total of 10 min. (A) Representative images showing basal, peak, and post peak fluorescence. Individual cell traces measured after (B) 24 and (D) 48 h peak net Ca^2+^ levels (after subtraction of basal levels) measured (C) 24 and (E) 48 h after LKE. Data are mean ± SE of 200 individual cells. *p < 0.05; **p < 0.005 versus same day control cells, unpaired *t*‐test. Scale bar is 80 μm.

**FIGURE 5:**
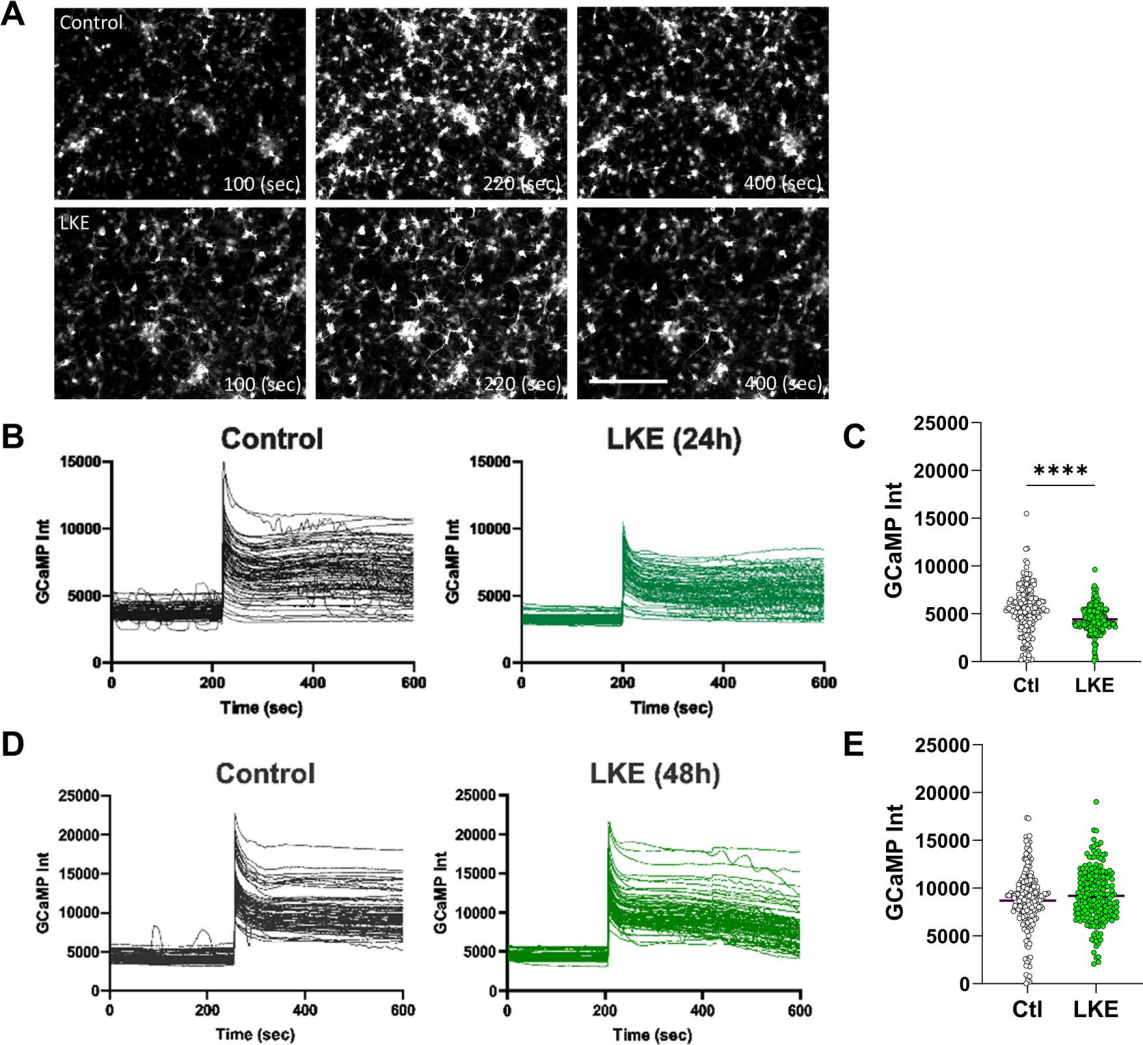
OPC Ca^2+^ responses to ATP are reduced by LKE. OPCs were infected with Ad‐GCaMP6 for 1 day, then incubated with 50 μM LKE for 24 or 48 h, and imaged before and after stimulation with 100 uM ATP. GCaMP images were obtained at 2 s intervals for a total of 10 min. (A) Representative images showing basal, peak, and post peak fluorescence. Individual cell traces measured after (B) 24 and (D) 48 h peak net Ca^2+^ levels (after subtraction of basal levels) levels measured (C) 24 and (E) 48 h after LKE. Data are mean ± SE of 200 individual cells. ****p < 0.0001 versus same day control cells, unpaired *t*‐test. Scale bar is 80 μm.

Stimulation with glutamate led to large increases in Ca^2+^ responses (Figure 4A), increasing by 2.6‐fold after 24 h and 2.4‐fold after 48 h (Figure 4B,D). However, in contrast to the effects of LKE on KCl induced Ca^2+^ responses, the increases due to glutamate were reduced in LKE‐treated cells (8% reduction at 24 h; and 10% reduction at 48 h, Figure 4C,E). Stimulation with ATP (Figure 5) caused similar increases of Ca^2+^ levels in control cells (2.5‐fold at 24 h; 3.2‐fold at 48 h) (Figure 5B,D), and similar to glutamate the increase at 24 h was reduced in LKE‐treated cells by 18% (Figure 5C), although not at 48 h (Figure 5E).

**FIGURE 6:**
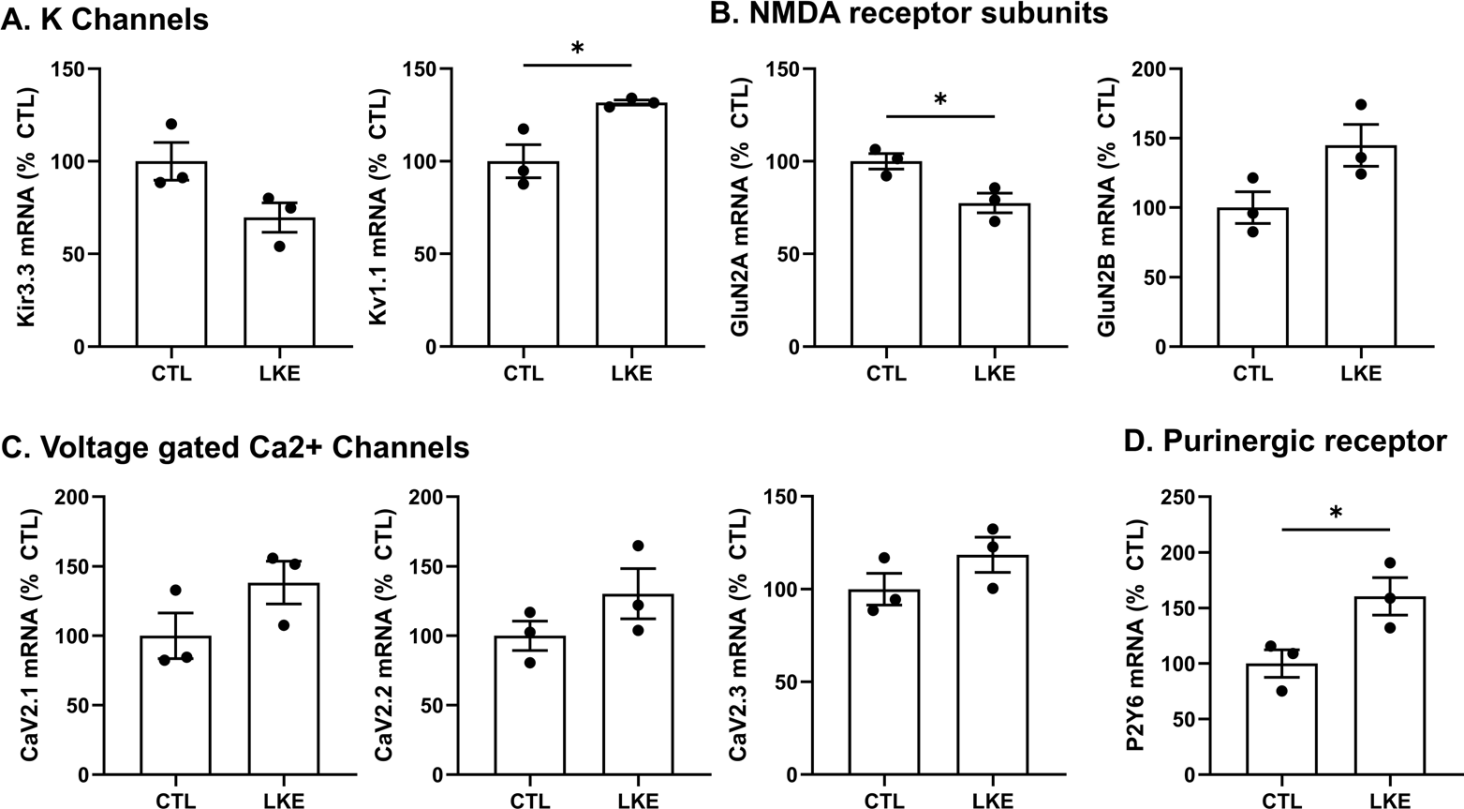
Effects of LKE on relative mRNA expression levels. Primary mouse OPCs were incubated with 10 μM LKE for 24 h, and then total mRNA was isolated and used for qPCR quantitation of (A) K channels Kir3.3 and Kv1.1; (B) NMDAR subunits GluN2A and GluN2B; (C) Voltage‐gated calcium channels CaV2.1, 2.2, and 2.3; and (D) the P2Y6 ATP purinoceptor. Data are mean ± SE of *n* = 3 samples per group, shown as % of control values. *p < 0.05, unpaired *t*‐test.

To determine if any LKE‐dependent changes in Ca^2+^ signaling were associated with changes in relevant channels or receptors, we quantified levels of relevant mRNAs measured after 24 h incubation. LKE significantly increased the relative mRNA levels of the potassium voltage‐gated channel Kv1.1 (Figure 6A), whereas levels of Kir3.3 (G‐protein activated inward rectifier potassium channel) were lower but not statistically significant. LKE significantly decreased levels of the NMDAR GluN2a subunit (Figure 6B), which could contribute to a reduced response to glutamate, whereas GluN2b levels tended to be higher. Measurement of VGCCs (Figure 6C) showed trends toward higher levels of the VGCCs CaV2.1, 2.2, and 2.3; however, those increases did not reach statistical significance. Interestingly, LKE significantly increased the P2Y6 purinoceptor mRNA (Figure 6D) suggesting that changes in this receptor do not mediate the reduced response to ATP.

**FIGURE 7:**
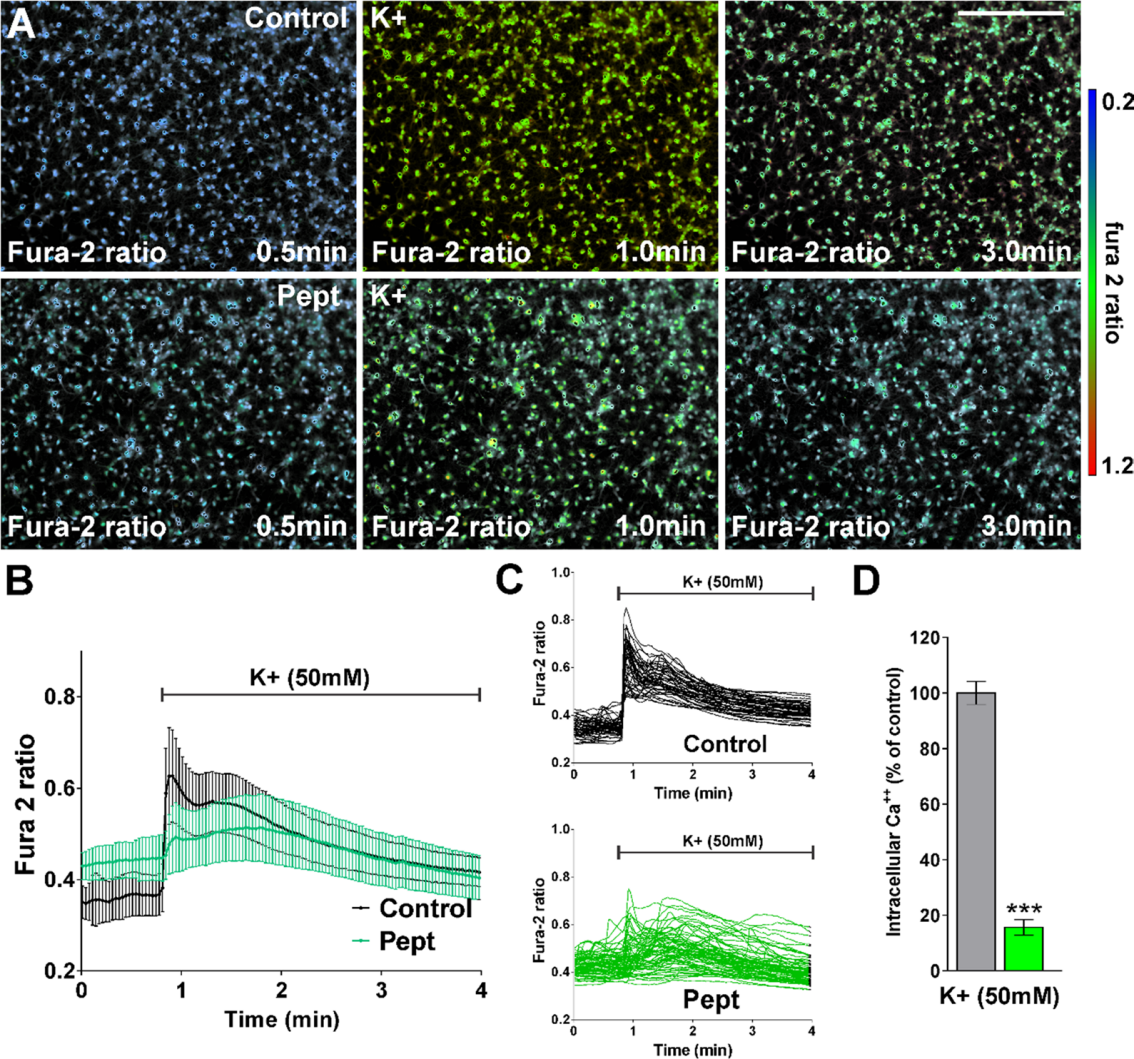
OPC Ca^2+^ responses to KCl are reduced by TAT‐CBD3 peptide. (A) Fura‐2 images were obtained at 2 s intervals for a total of 4 min. An increased fura‐2 fluorescence ratio is indicated by warmer colors. Scale bar = 120 μm. (B) Average Ca^2+^ responses in OPCs after high potassium stimulation (50 μM) (*n* ≥ 50 cells per condition). (C) Representative Ca^2+^ traces from individual OPCs are shown. (D) The bar graph shows the average amplitude of the Ca^2+^ response, calculated from the responding cells expressed as a percentage of change of the emission intensities. Values are expressed as mean ± SEM of at least four independent experiments. ***p < 0.001 versus control. Scale bar is 90 μm.

LKE has been shown to inhibit phosphorylation of CRMP2 and thereby modify its interactions with target partners including NMDAR subunits and VGCCs. To test if CRMP2 regulates Ca^2+^ influx, we incubated OPCs with TAT‐CBD3, a small peptide which disrupts CRMP2 interactions with other proteins, and then measured Ca^2+^ responses assessed by increases in the ratio of fura‐2 fluorescence signals. After 24 h incubation, basal Ca^2+^ levels were increased by TAT‐CBD3, going from a ratio of 0.28 to 0.45, an approximate 60% increase (Figure 7B,C). Following stimulation of control cells with KCl (Figure 7B,C) there was a rapid increase (from 0.35 to 0.65, an 1.8‐fold increase), followed by a slow decay. In cells incubated with peptide for 24 h, there was a gradual increase (from 0.44 to 0.50, a 15% increase) which then decayed, corresponding to an overall increase of Ca^2+^ approximately 15% of that of the control cells (Figure 7D).

**FIGURE 8:**
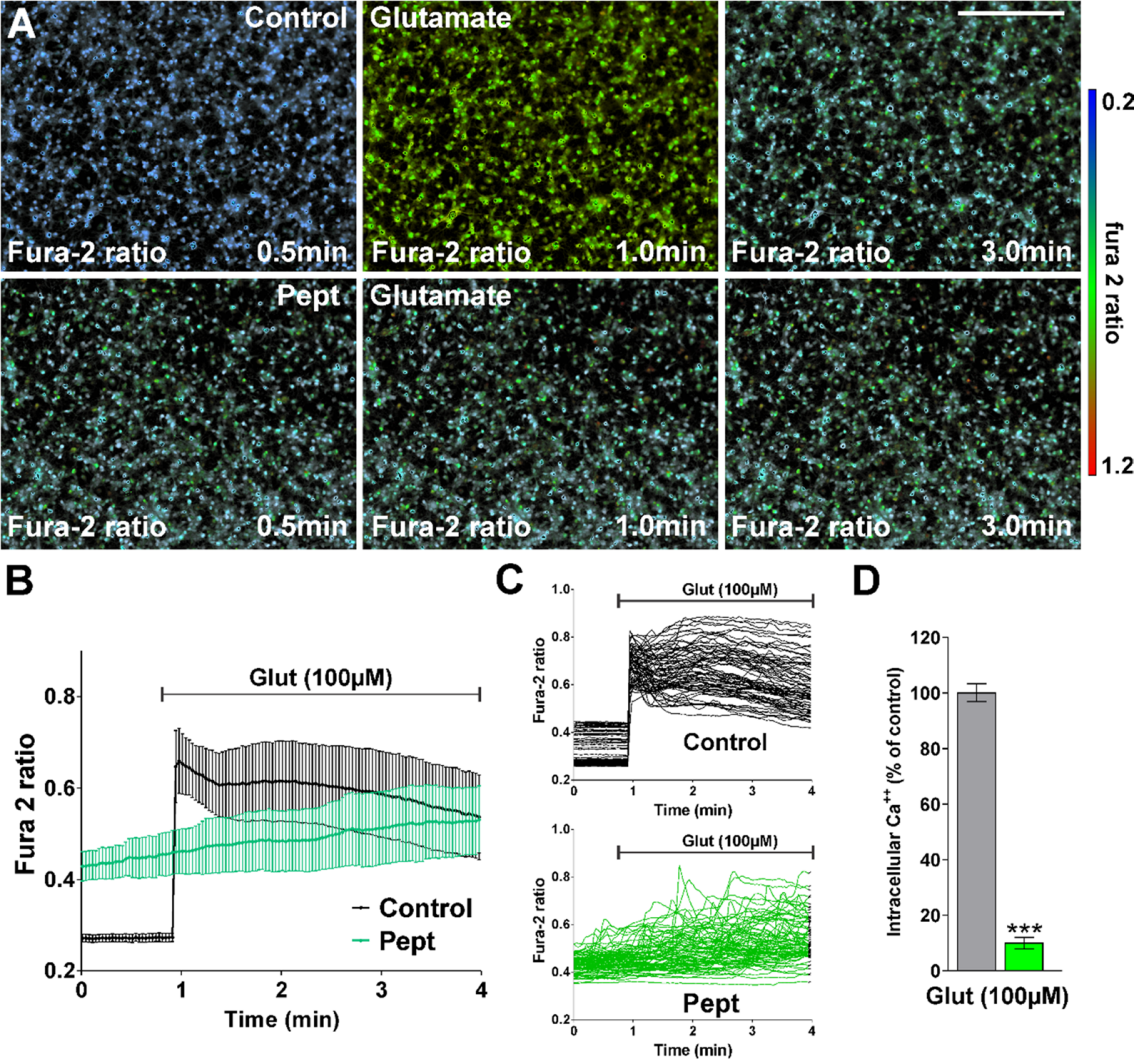
OPC Ca^2+^ responses to glutamate are reduced by TAT‐CBD3 peptide. (A) Fura‐2 images were obtained at 2 s intervals for a total of 4 min. An increased fura‐2 fluorescence ratio is indicated by warmer colors. Scale bar = 120 μm. (B) Average Ca^2+^ responses in OPCs after glutamate stimulation (100 μM) (*n* ≥ 50 cells per condition). (C) Representative Ca^2+^ traces from individual OPCs are shown. (D) The bar graph shows the average amplitude of the Ca^2+^ response, calculated from the responding cells expressed as a percentage of change of the emission intensities. Values are expressed as mean ± SEM of at least four independent experiments. ***p < 0.001 versus control. Scale bar is 90 μm.

**FIGURE 9:**
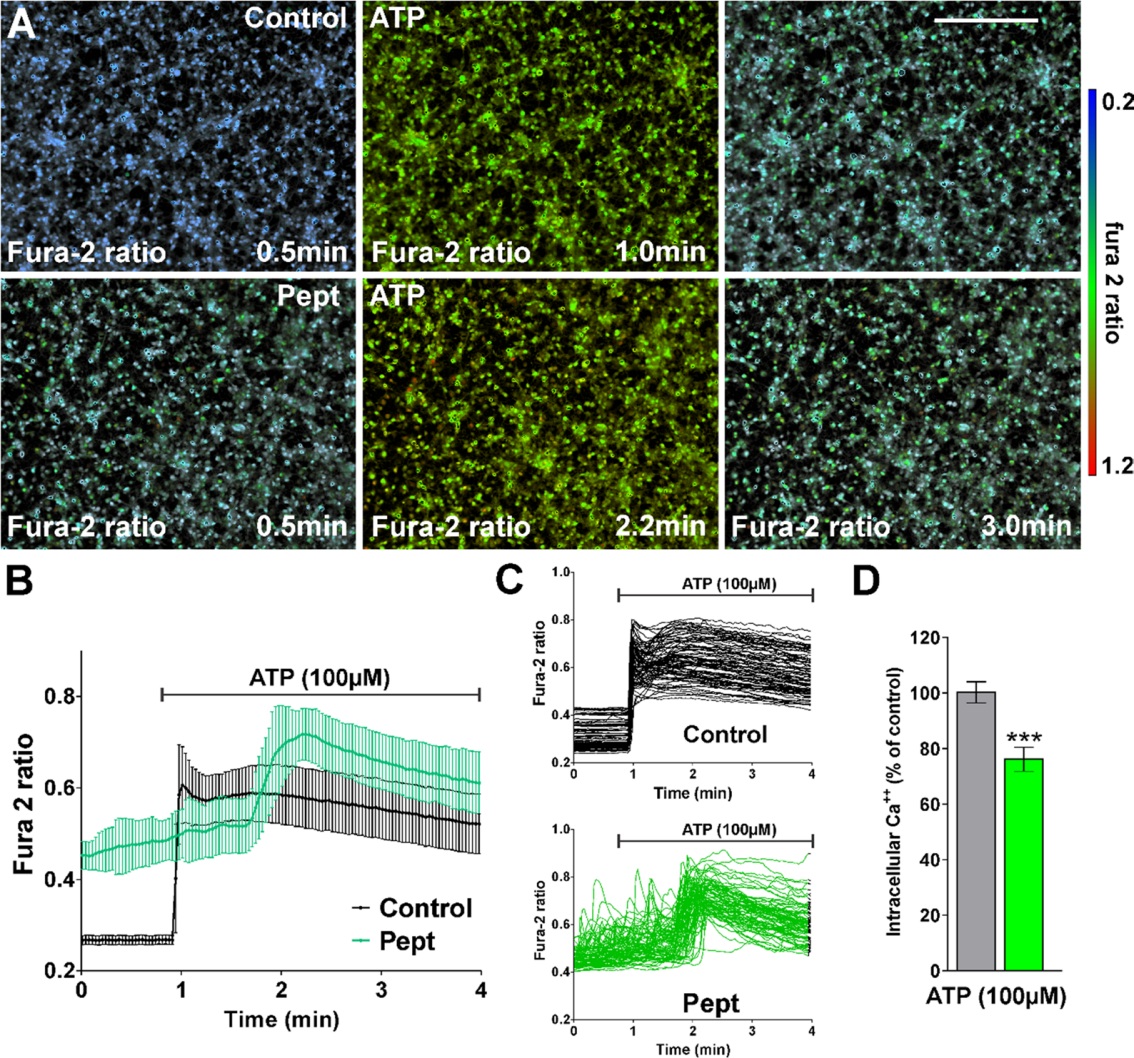
OPC Ca^2+^ responses to ATP are reduced by TAT‐CBD3 peptide. (A) Fura‐2 images were obtained at 2 s intervals for a total of 4 min. An increased fura‐2 fluorescence ratio is indicated by warmer colors. Scale bar = 120 μm. (B) Average Ca^2+^ responses in OPCs after ATP stimulation (100uM) (*n* ≥ 50 cells per condition). (C) Representative Ca^2+^ traces from individual OPCs are shown. (D) The bar graph shows the average amplitude of the Ca^2+^ response, calculated from the responding cells expressed as a percentage of change of the emission intensities. Values are expressed as mean ± SEM of at least four independent experiments. ***p < 0.001 versus control. Scale bar is 90 μm.

Following stimulation with glutamate (Figure 8), control cells showed a rapid increase of Ca^2+^ levels (from a fura‐2 ratio of 0.27 to 0.65, approximately a 2.5‐fold increase) which slowly decreased, whereas in the peptide‐treated cells, there was a gradual increase of Ca^2+^, leading to an average increase in the fura‐2 ratio from 0.42 to 0.52 (a 25% increase) after 3 min (Figure 8B,C), reflecting a 15% increase of intracellular Ca^2+^ compared to control cells (Figure 8D). Stimulation of control cells with ATP (Figure 9A) caused a rapid increase in the fura‐2 ratio (from 0.27 to 0.60, a 2.3‐fold increase) followed by slow decay; whereas in peptide‐treated cells, Ca^2+^ levels increased after a 1 min delay, increased from a fura‐2 ratio of 0.45 to 0.70 (a 1.6‐fold increase) and subsequently decayed (Figure 9B,C), reflecting an overall increase of intracellular Ca^2+^ levels approximately 75% of that of the control cells (Figure 9D).

## 4 Discussion

### 4.1 Effects of LKE on OPC Maturation and Proliferation

In the current study, we demonstrate that treatment with LKE exerts proliferative and differentiative actions on mouse OPCs. Incubation with LKE led to a significant increase in the number of Olig2+ expressing cells, as well as in expression of mature OLG markers CC1 and PLP. After 2 days, there was an increase of CC1+ and PLP+ stained cells, without any increase in Olig2, suggesting induction of maturation of pre‐existing Olig2+ cells. After 4 days, there were increases in CC1 and PLP as well as Olig2, suggesting an increase in both total cell numbers as well as further differentiation to mature OLGs. The percentage of Olig2+ cells that co‐expressed CC1 was increased by LKE from 18% to 65% after 2 days; and from 38% to 70% after 4 days. With respect to CC1+ cells that co‐express Olig2, LKE increased those from 55% to 78% on day 2; and from 64% to 91% on day 4. This raises the question as to the nature of CC1+ Olig2‐ cells. There are previous examples of OLG lineage cells that express CC1 but not Olig2 and were speculated to be mature OLGs that lost Olig2 expression (Payne et al. 2013). Those authors identified populations of CC1+/Olig2‐ cells that also co‐expressed Ki67, which increased during secondary degeneration following partial transection of the optic nerve, indicative of oligogenesis. The adenomatous polyposis coli (APC) protein can also be expressed in astrocytes (Etienne‐Manneville et al. 2003; Sakamoto et al. 2013), Bergmann glia (Bhat et al. 1996), and in the corpus callosum of cuprizone‐treated mice (Behrangi et al. 2021). Therefore the CC1+/Olig2‐ cells may be immature OPCs, post‐mitotic OLGs with low Olig2 expression, or a small population of astrocytes.

We also observed a decrease in caspase‐3 staining at both 2 and 4 days, suggesting that the effects of LKE may be due in part to reduced cell death. Measurement of cell proliferation, as assessed by Ki67 staining, shows that LKE increased OPC proliferation at both 2 and 4 days. We previously reported that following 5 weeks of cuprizone‐induced demyelination and remyelination carried out for 2 weeks in the presence of LKE did not alter the percentage of double‐labeled Olig2+:Ki67+ stained cells as compared to remyelination in the absence of LKE (Dupree et al. 2022). Differences with the current in vitro results may be because of the different treatment duration (2–4 days in vitro vs. 2 weeks in vivo), or to the induction of proliferation of Olig2‐negative cells present in our OPC cultures. However, overall these results demonstrate that LKE can induce both OPC proliferation as well as their maturation, which may account for its ability to accelerate remyelination in vivo.

It remains to be clarified how LKE can increase both proliferation and maturation. One possibility is that LKE has distinct effects on different OPC sub‐populations. For example, LKE could exert proliferative actions on immature OPCs but induce differentiation of more mature OPCs, both of which could be because of increased Ca^2+^ levels, or could reflect changes in expression of specific LKE targets. It is also possible that LKE only induces OPC maturation, and that the expanded pool of OPC subsequently matures.

### 4.2 Effects of LKE on Basal Ca^2+^ Levels

In view of reports that intracellular Ca^2+^ regulates various OPC functions including proliferation, differentiation, and myelin growth and remodeling (Paez and Lyons 2020), and that CRMP2, a primary target of LKE, has been shown to regulate Ca^2+^ flux (Brittain, Chen, et al. 2011; Brittain, Duarte, et al. 2011; Brittain et al. 2012; Feldman and Khanna 2013; Stratton et al. 2020), we tested if LKE would influence basal or stimulated Ca^2+^ levels. After 24 h treatment with LKE, there was no change in basal Ca^2+^ levels; however, after 48 h, basal Ca^2+^ levels were modestly (about 13%) increased by LKE. Under healthy conditions, intracellular Ca^2+^ levels regulate OPC maturation (Cheli et al. 2015), a modest increase in basal Ca^2+^ could promote OPC maturation and/or proliferation.

### 4.3 Effects of LKE on Stimulated Ca^2+^ Levels

Ca^2+^ entry into OPCs and OLGs is under regulation by a variety of channels and neurotransmitter receptors. During OLG maturation, intracellular Ca^2+^ is needed for development (Cheli et al. 2015; Paez and Lyons 2020); however, excess and sustained Ca^2+^ levels can contribute to cell damage. Normally, extracellular K^+^ levels, accumulated via release from neurons during signaling, are maintained by glial uptake of excess K^+^ through inward rectifier Kir4.1 channels and NaK ATPases, followed by spatial buffering via astrocytic gap junction networks. Increased neuronal firing, such as may occur under pathological conditions, or reduced K^+^ removal, for example, because of reduced astrocytic uptake mechanisms (Srivastava et al. 2020) can lead to significant increases in extracellular K^+^ and prolonged Ca^2+^ entry.

OLGs become depolarized in response to activation of OLG neurotransmitter receptors and an increase in intracellular K^+^ (Yamazaki et al. 2007) leading to Ca^2+^ influx through VGCCs (Barron and Kim 2019). VGCCs, particularly L‐type Ca^2+^ channels, are essential for normal OPC development (Cheli et al. 2016, 2015). During the postnatal development of the mouse brain, Ca^2+^ influx mediated by VGCCs promotes the survival and proliferation of immature OLGs (Cheli et al. 2016). Phosphorylation of proteins associated with these channels, as well as deletion of these channels, can interfere with OPC maturation, resulting in less expression of myelin proteins (Paez and Lyons 2020). Particularly, a gain‐of‐function mutation in Cav1.2 channels caused an increase in the proliferation of OPCs, facilitating their interactions with neurons through the activation of Ca^2+^/calmodulin‐dependent protein kinase‐II (Cheli et al., 2018). Our experiments demonstrate that LKE increases voltage‐gated Ca^2+^ signaling in OPCs. Although no changes were detected in the levels of Cav2.1, Cav2.2, or Cav2.3 mRNAs, LKE significantly enhanced Ca^2+^ influx in OPCs after plasma membrane depolarization with KCl. Thus, some of the effects of LKE on OPC proliferation and maturation could be mediated by increased activity of VGCCs.

Whether CRMP2 is involved in this response remains to be determined. Although primarily expressed in neurons in the adult brain, CRMP2 is also expressed in OLGs, and roles in OLG survival, maturation, and process extension have been described (Piaton et al. 2011; Syed et al. 2017). In addition to binding to tubulin, CRMP2 also interacts with VGCCs, targets Cav2.2 to neuronal membranes (Brittain et al. 2009), increases cell surface expression of VGCCs in CRMP2‐overexpressing neurons (Chi et al. 2009), and Cdk5‐mediated CRMP2 S522 phosphorylation increases association with Cav2.2, thereby increasing Ca^2+^ influx and neurotransmitter release (Chi et al. 2009). However, disruption of CRMP2:Cav2.2 interactions can be protective (Brittain et al. 2012), for example, under conditions of excessive Ca^2+^ influx leading to apoptosis. In this regard, some studies suggest that suppression of Cav2.2 is protective in EAE and MS. In MS lesions, treatment with omega‐conotoxin (a selective Cav2.2 inhibitor) reduced axon and myelin damage (Gadjanski et al. 2009), whereas in MOG peptide EAE, a1B null mice have reduced clinical signs and less demyelination (Tokuhara et al. 2010); and treatment with ziconotide‐a (selective Cav2.2 blocker) reduced clinical signs and neuroinflammation (Silva et al. 2018). Hence, an LKE‐dependent increase in Ca^2+^ in response to KCl could involve modulation of CRMP2:VGCC interactions.

### 4.4 Effects on Glutamate Responses

OPCs and OLGs express NMDARs, with several studies indicating that glutamate can activate NMDA‐mediated Ca^2+^ influx in both OPCs and mature OLGs (Káradóttir and Attwell 2007; Káradóttir et al. 2005). Importantly, NMDAR‐mediated current may be restricted to non‐proliferative OPCs that are in a quiescent state, or a state ready for differentiation into OLGs (Spitzer et al. 2019). The role of NMDARs in regulating OPC development has been more difficult to define. Cell type‐specific ablation of NMDAR function from the OLG lineage does not significantly affect OPC maturation and myelination (De Biase et al. 2011). Additionally, studies have shown that blocking NMDAR activity on OPCs does not drastically affect their rate of cell division, indicating that NMDAR signaling is not a primary driver of OPC proliferation (De Biase et al. 2011). However, LKE reduced the activity of NMDAR in primary neurons, preventing cell death because of glutamate ecotoxicity and the production of reactive oxygen species in these cells (Marangoni et al. 2018). Likewise, our Ca^2+^ imaging experiments performed in primary OPCs showed that LKE significantly reduced Ca^2+^ influx after glutamate stimulation. Additionally, we have detected a significant reduction in apoptotic cell death (caspase 3+ cells) in LKE‐treated OPCs. Therefore, it is possible that LKE's effect on glutamate signaling is promoting OPC survival in our culture conditions, stimulating indirectly the number of proliferating OPCs as well as the density of mature CC1‐expressing OLGs. Several studies have shown that CRMP2 interacts with NMDAR, as well as the Na+/Ca^2+^ exchanger, thereby regulating their functional activity and contributing to the control of Ca^2+^ levels within neurons (Brustovetsky et al. 2014). Thus, LKE's effects on glutamate signaling may also involve modulation of CRMP2.

CRMP2 also interacts with GluN2B‐containing NMDARs (Bretin et al. 2006; Moutal et al. 2014), and disruption of those interactions reduces NMDAR‐mediated currents, providing neuroprotection in models of brain injury (Brittain et al. 2012; Brustovetsky et al. 2014). NMDAR‐mediated damage occurs in white matter (Matute 2010); for example, in rat optic nerves, NMDARs mediate Ca^2+^ accumulation into myelin. Broad‐spectrum NMDAR antagonists (AP5, MK801) block those increases and reduce myelin damage (87), and NMDAR antagonists are protective in EAE (Dąbrowska‐Bouta et al. 2015; Farjam et al. 2014; Wallström et al. 1996). In OLGs, NMDARs are expressed on processes and in myelin (Matute 2011), which, upon activation, could contribute to excitotoxic cell death (Micu et al. 2006).

Examination of mRNA levels for receptors and channels that could mediate the effects of LKE on Ca^2+^ signaling shows that LKE significantly reduced expression of the NMDA GluN2A subunit (Figure 5B). This suggests that reduced Ca flux may be because of reduced expression of NR2A‐containing NMDARs. We previously showed that treatment of primary cerebellar granule cells with LKE reduced cell death because of glutamate (Marangoni et al. 2018), and was associated with reduced levels of reactive oxygen species. Although interactions of LKE with NMDARs have not been reported, several studies have demonstrated interactions between CRMP2 and NMDAR subunits. In one study, it was shown that both NMDAR subunits NR2A and NR2B co‐precipitated with CRMP2 from hippocampal membrane fractions using antibodies directed to NR2A or NR2B, although stronger binding was observed with NR2B (Al‐Hallaq et al. 2007).

### 4.5 Effects on ATP Responses

Whereas glutamate has been shown to regulate mostly the early development of OLGs, adenosine and ATP are modulators of late OLG development and myelination. Similar to glutamate and K+, ATP and adenosine are released by electrically active axons of the CNS. It has been shown that adenosine activates receptors on OPCs, which increases intracellular Ca^2+^ concentration, reducing OPC proliferation and stimulating the migration of these cells (Othman et al. 2003; Stevens et al. 2002). Thus, a reduction in Ca^2+^ influx mediated by adenosine and/or ATP can potentially promote OPC proliferation. We found that 24 h treatment with LKE significantly reduced OPC Ca^2+^ influx in response to ATP. Although this effect disappeared after 48 h of LKE treatment, transient reductions in adenosine and ATP channel activities could profoundly influence the rate of OPC division.

### 4.6 Effects of TAT‐CBD3 on Ca^2+^ Flux

Although the exact targets of LKE have not been fully defined, proteomics analyses identified CRMP2 as a major synaptic protein that interacts with LKE, and several studies demonstrated that the non‐phosphorylated form of CRMP2 binds to microtubules and stabilizes neuronal processes (Hensley and Kursula 2016). LKE prevents CRMP2 phosphorylation at the serine 522 site, thus promoting synaptogenesis, neuronal spine growth, and axonal growth (Zhang et al. 2018). It is therefore possible that regulation of CRMP2 phosphorylation by LKE also stabilizes the microtubule network at OPC processes, thereby promoting morphological maturation of these cells and expression of myelin proteins such as PLP.

To better determine if the above interactions of CRMP2 with VGCCs or NMDARs influence OPC proliferation or maturation, we tested if blocking those interactions with a small molecular weight peptide would replicate effects of LKE on Ca^2+^ flux. TAT‐CBD3 is a 15 amino acid peptide derived from the calcium binding domain 3 of CRMP2 (Khanna et al. 2020). TAT‐CBD3 and other modified forms of CBD3 have been shown to reduce NMDAR‐mediated Ca^2+^ influx and damage in neurons (Brittain, Chen, et al. 2011; Brittain et al. 2012; Brustovetsky et al. 2014). These peptides also disrupt the interaction of CRMP2 with the N‐type CaV2.2 VGCC (Brittain, Duarte, et al. 2011) and by doing so reduce Ca^2+^ dependent neuropeptide release, alleviating neuropathic pain (Perez‐Miller et al. 2024; Wilson et al. 2011). As found with LKE treatment, following 24 h incubation with the TAT‐CBD3 peptide, there was an approximately 60% increase in basal Ca^2+^ levels. Likewise, Ca^2+^ responses to glutamate and ATP were also reduced in TAT‐CBD3 treated cells. In contrast to LKE, the peptide led to a decrease in the Ca^2+^ response to depolarization by KCl (Figure 7). Together, these findings suggest that both LKE and the TAT‐CBD3 peptide could provide protection to OPCs upon exposure to increased extracellular levels of glutamate or ATP, and that LKE's effect on depolarization may not be mediated via CRMP2.

## 5 Conclusions

Our findings demonstrate proliferative and differentiation effects of LKE in primary cultures of mouse OPCs, associated with changes in Ca^2+^ responses to depolarization, glutamate, and ATP. mRNA analysis suggests that the reduced glutamate responses may be mediated in part by NR2a expressing NMDARs; however, further analysis is needed to measure mRNA and protein levels of other NMDAR subunits and if other changes occur after longer periods of incubation. Treatment of OPCs with the TAT‐CBD3 replicated some of LKE actions, pointing to a role for CRMP2; however, direct studies to test CRMP2 involvement, for example, using CRMP2 depleted cells, are needed to confirm those results. Finally, in vivo studies have demonstrated beneficial actions of LKE in mouse models of MS; whether those are also accompanied by modulation of Ca^2+^ responses remains to be determined. Overall, these findings suggest that therapeutic interventions that target Ca^2+^ flux in OPCs may provide benefit for demyelinating diseases.

### Declaration of Transparency

The authors, reviewers and editors affirm that in accordance to the policies set by the *Journal of Neuroscience Research*, this manuscript presents an accurate and transparent account of the study being reported and that all critical details describing the methods and results are present.

## Author Contributions

D.L.F., J.L.D., P.M.P., and T.T.D. conceptualized the study. T.T.D. synthesized and purified LKE. V.T.C., S.G.T., and Z.M. carried out experiments, collected, and analyzed data. V.T.C., P.M.P., and D.L.F. generated figures and tables. J.L.D., P.M.P., and D.L.F. wrote and edited the manuscript.

## Ethics Statement

All animal studies were approved by the Institution Animal Care and Use Committee at the University of Illinois, University of Buffalo, and McGuire VA.

## Conflicts of Interest

The authors declare no conflicts of interest.

## Peer Review

The peer review history for this article is available at https://www.webofscience.com/api/gateway/wos/peer‐review/10.1002/jnr.70061.

## Supporting information


**Data S1.** Transparent Science Questionnaire for Authors

## Data Availability

The data that support the findings of this study are available on request from the corresponding author. The data are not publicly available because of privacy or ethical restrictions.
